# Transdisciplinary Research Priorities for Human and Planetary Health in the Context of the 2030 Agenda for Sustainable Development

**DOI:** 10.3390/ijerph17238890

**Published:** 2020-11-30

**Authors:** Kristie L. Ebi, Frances Harris, Giles B. Sioen, Chadia Wannous, Assaf Anyamba, Peng Bi, Melanie Boeckmann, Kathryn Bowen, Guéladio Cissé, Purnamita Dasgupta, Gabriel O. Dida, Alexandros Gasparatos, Franz Gatzweiler, Firouzeh Javadi, Sakiko Kanbara, Brama Kone, Bruce Maycock, Andy Morse, Takahiro Murakami, Adetoun Mustapha, Montira Pongsiri, Gerardo Suzán, Chiho Watanabe, Anthony Capon

**Affiliations:** 1 Center for Health and the Global Environment (CHanGE), University of Washington, Seattle, WA 98195, USA; 2 University of Hertfordshire, Hatfield AL10 9AB, UK; F.harris@herts.ac.uk; 3 Future Earth, Global Hub Japan, Tsukuba 305-0053, Japan; gilessioen@s.k.u-tokyo.ac.jp; 4 National Institute for Environmental Studies, Tsukuba 305-0053, Japan; chiho.watanabe@nies.go.jp; 5 Towards A Safer World Network (TASW), 16561 Stockholm, Sweden; cwannous@yahoo.com; 6 Biospheric Sciences Laboratory, NASA Goddard Space Flight Center, Universities Space Research Association, Greenbelt, MD 20771, USA; assaf.anyamba@nasa.gov; 7 School of Public Health, The University of Adelaide, Adelaide 5005, Australia; peng.bi@adelaide.edu.au; 8 Department of Environment and Health, School of Public Health, Bielefeld University, 33615 Bielefeld, Germany; boeckmannmelanie@gmail.com; 9 Institute for Advanced Sustainability Studies, 14467 Potsdam, Germany; kathrynjbowen@gmail.com; 10 School of Population and Global Health, University of Melbourne, Melbourne 3052, Australia; 11 Fenner School of Environment and Society, Australian National University, Canberra 0200, Australia; 12 Swiss Tropical and Public Health Institute, University of Basel, CH-4002 Basel, Switzerland; gueladio.cisse@swisstph.ch; 13 University of Basel, CH-4001 Basel, Switzerland; 14 Institute of Economic Growth, Delhi 110067, India; purnamita.dasgupta@gmail.com; 15 Department of Health Systems Management and Public Health, The Technical University of Kenya, Nairobi, Kenya; gdidah@gmail.com; 16 School of Public Health and Community Development, Maseno University, Private Bag 40100, Kisumu, Kenya; 17 Institute For Future Initiatives, The University of Tokyo, Tokyo 113-0033, Japan; gasparatos.alex@gmail.com; 18 Global Interdisciplinary Science Programme on Urban Health and Wellbeing: A Systems Approach, Institute of Urban Environment, Chinese Academy of Sciences, Xiamen 361021, China; franz@iue.ac.cn; 19 Institute of Decision Science for a Sustainable Society, Kyushu University, Fukuoka 819-0395, Japan; javadi.firouzeh.467@m.kyushu-u.ac.jp (F.J.); murakami.takahiro.023@m.kyushu-u.ac.jp (T.M.); 20 Disaster Nursing Global Leadership Program, University of Kochi, Kochi 781-8515, Japan; kanbara@cc.u-kochi.ac.jp; 21 Lecturer-Researcher of Public Health, University Peleforo Gon Coulibaly of Korhogo, Korhogo, Cote D′Ivoire; brama.kone@csrs.ci; 22 Centre Suisse de Recherches Scientifiques in Côte d’Ivoire, Abidjan, Cote D′Ivoire; 23 College of Medicine & Health, University of Exeter, Cornwall TR1 3HD, UK; bmaycock@iinet.net.au; 24 School of Environmental Sciences, University of Liverpool, Liverpool L69 3GP, UK; A.P.Morse@liv.ac.uk; 25 Nigerian Institute for Medical Research, 6 Edmund Crescent, Yaba, Lagos, Nigeria; adetoun.mustapha03@alumni.imperial.ac.uk; 26 Stockholm Environment Institute, Asia Centre, Bangkok 10330, Thailand; mpongsiri@gmail.com; 27 Facultad de Medicina Veterinaria y Zootecnia, Universidad Nacional Autónoma de México, Mexico City 03100, Mexico; gerardosuz@gmail.com; 28 Monash Sustainable Development Institute, Monash University, Melbourne 3800, Australia; tony.capon@monash.edu

**Keywords:** adaptation, biodiversity, climate, ecosystems, health, land use, mitigation, oceans, risk management

## Abstract

Human health and wellbeing and the health of the biosphere are inextricably linked. The state of Earth’s life-support systems, including freshwater, oceans, land, biodiversity, atmosphere, and climate, affect human health. At the same time, human activities are adversely affecting natural systems. This review paper is the outcome of an interdisciplinary workshop under the auspices of the Future Earth Health Knowledge Action Network (Health KAN). It outlines a research agenda to address cross-cutting knowledge gaps to further understanding and management of the health risks of these global environmental changes through an expert consultation and review process. The research agenda has four main themes: (1) risk identification and management (including related to water, hygiene, sanitation, and waste management); food production and consumption; oceans; and extreme weather events and climate change. (2) Strengthening climate-resilient health systems; (3) Monitoring, surveillance, and evaluation; and (4) risk communication. Research approaches need to be transdisciplinary, multi-scalar, inclusive, equitable, and broadly communicated. Promoting resilient and sustainable development are critical for achieving human and planetary health.

## 1. Introduction

The Anthropocene epoch is underway; human activities are profoundly changing the earth system [1,2]. Global environmental changes, including biodiversity loss, the nitrogen cycle, and climate change, are exceeding the Earth’s planetary boundaries [3,4], with tipping points in multiple systems possible over the coming decades [5]. The field of planetary health recognizes that our lives, livelihoods, and societies rely upon the health of our planet. Climate change profoundly affects our health and wellbeing through, for example, extreme weather and climate events, the spread of infectious diseases, decreasing crop yields, and ecosystem degradation [6]. Further health challenges arise from other types of environmental change, including pollution of air, land-use change, and water pollution.

There is a mismatch between the growth in understanding of the magnitude and patterns of health risks associated with global environmental change and the speed of research and technology development into understanding recent and projecting future changes in the Earth system [2]. Research investments continue to be inadequate across all countries, impeding information on both understanding of current impacts and projected risks, and on the evidence-based policies and programs needed to prepare for and manage changing burdens of disease [7,8]. The COVID-19 pandemic dramatically highlights the challenges to and vulnerabilities of population health and health systems and highlights the value of systems-based approaches for risk management that integrate across sectors.

This review identifies how socioeconomic transitions and environmental change intersect with human and planetary health and identifies priority research areas. The main themes were identified through a participatory workshop under the auspices of the Future Earth Health Knowledge Action Network (Health KAN) held in May 2019 [9]. See Appendix A describing the background of the Health KAN. We (1) discuss the intersection of planetary health with socioeconomic transitions and environmental change (Section 3 and Section 4); (2) identify research priorities in these intersections (Section 5); and (3) identify characteristics of the research approaches needed to provide useful and fit-for-purpose research to inform, develop, implement, and evaluate interventions to improve sustainable development for planetary health and human well-being (Section 6).

## 2. Materials and Methods

Development of the research agenda was initiated at the Health KAN launch event held on 20–23 May 2019 at Academia Sinica, Taipei City; the participants are listed in the Annex. Invitations to participate in the workshop were widely distributed online and through personalized e-mails on 25 January 2019 across the Health KAN Open Network Membership (*N* = 210 members on 16 April 2019). The Open Network was an online community facilitated by Future Earth that includes members from diverse sectors who collaborate around the linkages between the environment and health; this which was recently changed to a Members’ Platform (can be accessed at https://members.futureearth.org/). Recipients of the email were informed that funding was available for some participants from the global South. Our objective was to ensure a diverse and representative range of participants and yet keep the group small enough for productive discussions. Of the 52 potential participants selected, 42 were able to attend (see Table A1 in Appendix B for the full list of participants). The participants consisted of 18 females and 24 males, the majority of whom were full-time academics (*n* = 25). Some were academics with an additional position in a governmental, intergovernmental, private, or non-profit organization (*n* = 8), some had their main occupation in a (inter)governmental organization, including funders (*n* = 5) and non-profit organizations (*n* = 4).

**Figure 1:**
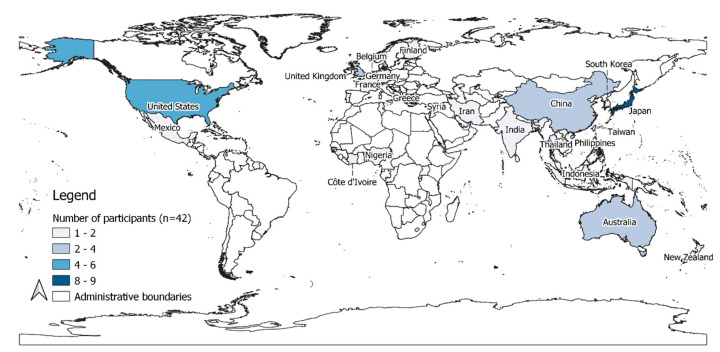
Participants (*n* = 42) by country of nationality.

Figure 1 shows the nationalities of the participants. South America was the only major populated continent that was not represented, although several participants were familiar with the research needs and had ongoing relevant projects there. The participants co-designed the agenda with the development team, including drafting a research agenda, under the leadership of the co-chairs and the oversight of the Future Earth secretariat.

**Figure 2:**
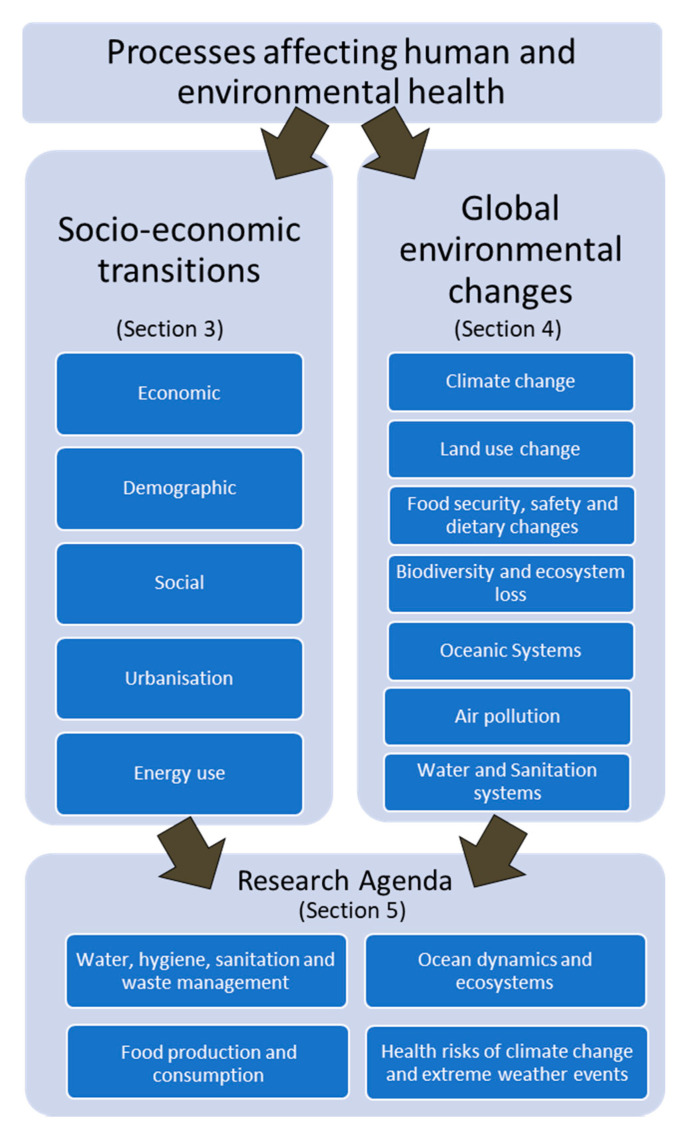
The Future Earth Health Knowledge Action Network (Health KAN) research agenda categorization.

Sessions dedicated to setting the research agenda followed presentations and discussions designed to strengthen collaboration for integrating sustainability research and innovation across sectors. The discussions were informed by an earlier research agenda proposed by the Health KAN development team and built on the UN Sustainable Development Goal (SDG) 3 that focuses on ensuring healthy lives and promoting well-being for all at every age. SDG 3 recognizes that despite progress in global health over the last decades, there continues to be significant, preventable morbidity and mortality worldwide. Much of this health burden is associated with environmental factors that are expected to change over the coming decades with changes in our climate, biodiversity, land use, and other factors. Following the launch event, teams were formed with 2–4 members to write a description for each research agenda category; these were reviewed and further refined by all workshop participants. The results of this are described in Section 3, Section 4 and Section 5 below as shown in Figure 2. We identified five processes of socioeconomic transition and seven processes of global environmental change that impact human and planetary health. Four broad areas would benefit from further research. Although presented as individual research topics, we recognize that these are not discrete, but rather interconnected topics, with potentially synergistic benefits (e.g., ocean dynamics, ecosystems, and food production).

## 3. Socioeconomic Transitions Affecting Human and Planetary Health

Socioeconomic changes affect human health and well-being. Processes that frame and shape the relationships between people and their environment, with subsequent impacts on global health, include how societies function and organize themselves, use land and natural resources, and manage health, water, and sanitation. This section briefly discusses socioeconomic transitions, followed by a discussion of global environmental changes affecting health. These are used to inform the grand research challenges in Section 5.

### 3.1. Economic

Economic growth, associated with increasing incomes, along with fundamental changes in the way society has been organized [10], has enabled transitions in living standards that have, in general, improved life expectancy, health status, poverty alleviation, and health care [11,12,13]. Economic growth also has led to environmental degradation with adverse population health consequences. The economic transition varied markedly over time, with differential progress across and within countries. Examples include ozone-damaging compounds that increase the likelihood of skin cancer and eye cataracts and the health impacts of air, water, and ocean pollution [14].

Increasingly, concerns for equity and equality within and across countries are overshadowing economic transitions. These inequities [15] can lead to the erosion of health systems and environmental and labor laws and regulations [16,17,18].

The COVID-19 pandemic has dramatically highlighted how economic well-being is intricately linked with planetary health and underscored the need for scientifically informed policies. The conventional belief that a successful economic transition can provide the resources needed for good health, longevity, and personal safety has been challenged. Failing to mainstream the relationship between economy and nature and health in decision-making can lead to severe economic stress over shorter and longer time periods. Organized collective management of the global commons is essential for future development and sustainability [10,19]). The COVID-19 pandemic highlights the value of research that recognizes and prioritizes the interdependencies between economic growth and global risks such as how climate change and biodiversity loss can affect human health and the capability to achieve resilient health and wellbeing [20].

### 3.2. Demographic and Social

There is a two-way process between increased economic growth and demographic transition: the demographic transition (e.g., drop in mortality) followed an increase in incomes, which facilitated economic growth (e.g., relatively higher investments in education and health enhanced the “quality” of a smaller population) [21,22]. Better nutrition and a lower disease rate are associated with a decline in fertility and an increase in formal education, along with a rise in adult longevity [23].

The demographic patterns projected over the next three decades indicate that research needs are multidimensional. It is critical for all countries to identify, implement, and institutionalize enabling conditions for green and blue technologies that minimize greenhouse gas emissions and air pollution while providing energy for sustainable production and consumption. Similarly, there is a continuing need for research on sustainable agriculture that leads to affordable and accessible food for meeting the nutritional needs of all while reducing waste and minimizing the degradation of natural resources.

### 3.3. Energy Systems

Despite positive consequences for human development, the energy strategy that brought the Anthropocene-massive combustion of fossil fuels-created a host of negative health, environmental, and economic outcomes [24,25]. Fossil fuel use for power generation, space heating, and transport are responsible for most recent climate change, in addition to releasing atmospheric pollutants ranging from gases to particulate matter [26,27,28]. Air pollutants associated with fossil fuel burning have significant negative consequences for human health [29].

Less expensive fuel options are generally less efficient, producing more smoke, and are often used in poorly designed homes [30]. For people without access to modern energy sources, cooking and lighting fuel cost a large portion of their income, increasing poverty and limiting opportunities for development [31]. Air pollution also is a risk factor for COVID-19.

The Paris Agreement committed signatories to a renewable energy transition to stabilize greenhouse gas emissions (UN Paris Agreement 2015). There are near immediate health benefits from reductions in exposure to air pollution from point sources such as coal-fired power plants and from mobile sources such as transport [32,33]. The magnitude of these health benefits is of the same order as the costs of mitigation.

Innovations are needed to improve access to clean and reliable energy through off-grid electrification solutions, expansion of renewable generation capacity, improvement of service efficiency, etc. These innovations need to be researched to ensure they also reduce the burden of diseases associated with household air pollution, including premature deaths and fatal accidents.

### 3.4. Urban Systems

The world is in the midst of a global urban transition. In the past 200 years, the proportion of the world’s population living in cities grew from about 5% to more than 50% [34]. By 2050, nearly 70 out of 100 people worldwide will live in cities, with more than 1 million people moving into cities every week; 90% of the increase will be in Asia and Africa [35]. Cities are responsible for 85% of global economic activities and approximately 75% of greenhouse gas emissions [36]. The effects of urban living on health and wellbeing vary widely and are affected by wealth, social status, and specific features of the urban environment [37]. In high- and middle-income countries, urban health threats include air and water pollution, noise, barriers to physical activity, absence of green space, and in some cases social exclusion, poverty, and unaffordable housing. Health challenges in cities include cardiovascular disease, cancers, diabetes, and mental health problems. Cities in low-income countries experience these problems, along with critical shortages of infrastructure (potable water, sanitation, electricity, waste management, transport), inadequate housing, uncertain land tenure, food and nutritional insecurity, poor governance, and other challenges [38,39,40,41]. Additional challenges include vector-borne and waterborne diseases and pollution-related health problems.

To date, the COVID-19 pandemic has been more severe in cities, highlighting the interlinkages of social and economic systems on urban health [42]. Reducing the associated risks [43], from urban health and financial emergencies, for example, will substantially improve health and wellbeing. This underlines the importance of a better understanding of how cities work as complex adaptive systems and as hubs of interconnectivity. Innovations and solutions to overcoming systemic risks are most likely to originate in cities.

Urban heat islands will intensify with climate change [44]; the built environment will be an important mediating factor. Green space can reduce the urban heat island. Passive and active cooling of buildings may reduce the health effects of extreme heat, but more research is needed to determine cost-effective adaptations in different settings. Further, urban centers in coastal areas are often subject to storm surges, floods, and sea-level rise that may affect precarious and aging infrastructures, with the challenges expected to increase with further climate change [45,46]. An important research priority is understanding these vulnerabilities and the likely effectiveness of interventions to build resilience to acute and chronic stresses.

Recent analyses of the causal pathways from an urban health perspective found at least 48 SDG targets (of 169) relevant to urban health, mainly captured by SDG 3 (Health and wellbeing) and SDG11 (inclusive, safe, resilient, and sustainable cities). However, physical activity, noise pollution, quality of life, and social capital were not included in the SDGs [47]. Filling these knowledge gaps with transdisciplinary research would benefit urban political leaders, urban planners, civil society, public health professionals, developers and builders, and other stakeholders.

## 4. Global Environmental Changes Affecting Human and Planetary Health

### 4.1. Climate Change

The frequency and intensity of extreme weather events are increasing with around 90% of disasters weather- or climate-related. The Emergency Events Database (EM-DAT) records a disaster when at least one of the following criteria are fulfilled: ten (10) or more people reported killed; hundred (100) or more people reported affected; declaration of a state of emergency; call for international assistance. Over the period 2008–2017, 84.2% of the 3751 natural hazards recorded in EM-DAT were weather-related (e.g., heatwaves, floods, storms, and droughts), resulting in 2 billion people affected and economic losses of an estimated 1658 billion US dollars [48]. These events cause acute injuries and deaths, which may be followed by cascading impacts affecting health and well-being, including infectious diseases, mental illness, hunger, conflict, and population mobility. Extreme events also can damage and destroy health physical infrastructure, resulting in hospital closures, compounding health challenges.

Climate change also is altering environmental exposures that can negatively affect health, including changing patterns of respiratory diseases associated with air pollution (e.g., ozone and aeroallergens), vector-, food-, and water-borne diseases, and through socially mediated effects that can result in population displacement, risks of violent conflict, and slowing of economic growth and poverty reduction. Climate change also could alter the magnitude and pattern of non-communicable diseases through altering work-related injuries and illnesses and by affecting the productivity of outdoor workers. Projections indicate that for most health outcomes, each additional unit of warming will be associated with higher risks. By 2030, climate change and natural disasters may push 77 million more urban residents into poverty [49,50].

The risk of disease outbreaks is associated with population size, health status, and living conditions. Crowding, inadequate water and sanitation, and poor access to health services increase the risk of communicable disease transmission [51]. Often these effects are complex and interrelated, examples include the outbreak of cholera in East Africa associated with the 2015–2016 El Niño [52]; and the association of dengue with elevated temperatures throughout Southeast Asia in 1997–1998.

### 4.2. Land Use

Large-scale land-use change is one of the great transitions of the Anthropocene, in particular, the conversion of primary forest to agriculture and then through intensification to degraded land, with over 75% of global land cover anthropogenically degraded [2,53,54]. Continued anthropogenic pressure on remnant intact forests and increasing agricultural intensification and urbanization are expected [2,53,54]. The drivers of this transition are economic development (e.g., global food production) and economic growth (e.g., investment in physical capital like infrastructure). While there are significant health benefits to these drivers, the negative effects of land-use change on human health and welfare are underestimated and poorly accounted for. Conversion of forested land to agricultural use has been the main driver of deforestation, leading to the loss of key ecosystem services such as carbon storage and sequestration, biodiversity, water, and regulation of climate and diseases [54]. The underlying factors affecting forest conversion include population growth, agricultural development, land tenure, governance of land-use changes, changing markets, technological improvements, and active policy interventions. Overall, the economic global losses of ecosystem services due to land conversion are approximately $20 billion to $9.4 trillion USD per year [55,56].

These losses underestimate or fail to account for negative health outcomes due to the process of land clearance, or the consequences of altering animal-human-microbial interactions across landscapes, both of which are likely widespread and globally significant. Around 31% of the zoonotic infectious diseases that emerged since 1940 are associated with some types of land conversion, including fragmentation, agricultural intensification, and deforestation, leading to millions of deaths and substantial loss of global labor productivity [57] (see Reference [58] Lyme disease in NE US; see Reference [59] malaria, *Anopheles darlingi* in Peruvian Amazon).

### 4.3. Food Safety and Security, and Dietary Changes

Ensuring food security and adequate nutrition without compromising planetary health will be one of the global grand challenges for the 21st century [60]. After declining for several years, global hunger increased from 804 million in 2016 to more than 820 million people in 2018 (one out of every nine people) [61]. Over two billion people globally do not have regular access to safe, nutritious, and sufficient food. At the same time, about 144 million children under five were stunted and 38 million were overweight in 2019, with high rates of malnutrition, including undernutrition and overnutrition, coexisting in many countries [62]. These impacts are disproportionately felt in low- and middle-income countries.

Diets and dietary preferences are major elements of food systems [63], which are changing with rising affluence, e.g., more people consuming larger quantities of meat and dairy products, driving a market for livestock products and impacting the environment (see below) [64]. Steering diets and dietary preferences (and their change) toward more sustainable pathways will be essential for achieving sustainability and enhancing planetary health [60].

The food system (e.g., the web of activities involving production, processing, transport, consumption, and waste of food) has huge environmental impacts (which are expected to increase), including greenhouse gas emissions, land-use change for agriculture, biodiversity loss (including pollinator loss), fisheries depletion, soil degradation, effects of fertilizers and pesticides, pollution, and freshwater depletion [2,65,66,67,68].

At the same time, climate change threatens food security through its impacts on agriculture, food, health, and socio-demographic and economic systems. The impacts of climate change on agricultural yields, livestock productivity, the prevalence of pests and diseases, food prices, and food safety/quality are projected to have major implications for sustainable development, inequality, poverty eradication, and the achievement of the Sustainable Development Goals (SDGs). Limiting warming to 1.5 °C is projected to result in smaller net reductions in yields of maize, rice, wheat, and potentially other cereal crops, particularly in sub-Saharan Africa, Southeast Asia, and Central and South America, and in the CO_2_-dependent nutritional quality of rice and wheat [69].

### 4.4. Biodiversity and Ecosystem Service Loss

Many cultures rely on a mixture of agriculture, hunting, and gathering natural products for food. Diversity of species and gene pools can increase the productivity of farming systems in a range of growing conditions, and more diverse farming systems are also generally more resilient in the face of perturbations, thus enhancing food security. Even populations that are predominantly dependent on intensive agriculture may benefit from biodiversity for wellbeing because of crop productivity increases in biodiverse systems [70].

In the last 100 years, more than 90% of crop varieties were lost in favor of genetically improved, high-yielding varieties. Many livestock breeds are at risk of extinction and the vast majority of the world’s food resources consists of only 12 plant and 5 animal species [71]. Crop diversity has been depleted and genetic similarity between modern crop cultivars increased [72]. This reduced diversity among crop species reduces the adaptation opportunities in the context of climate change. Narrowing the genetic diversity of crops creates risks to health and household food security, especially in rural communities [73].

Biodiversity provides significant benefits for health. About 60% of available drugs are either directly or indirectly derived from natural products [74]. Moreover, natural products have been an invaluable source of inspiration for organic chemists to synthesize novel drug candidates, as shown in Reference [75]. Some have claimed that the switch away from natural products to combinatorial chemistry during the 1990s may have led to the current paucity of new drug candidates in the development pipeline [76].

There is increasing evidence that for many host–pathogen systems, biodiversity loss drives increased the risk of disease transmission to people. Several studies proposed a “dilution effect hypothesis”, by which communities with high host diversity lower microbial transmission through an increased proportion of host species that are poor transmitters of specific microbes [77,78]. The dilution effect might be due to a high number of host species incapable of pathogen transmission, hence “blocking” the transmission pathway [79,80] and/or to inter-specific host competition in highly diverse communities that decrease the abundance of other host species [81,82]. There is evidence of a dilution effect for zoonotic infections, including viruses like hantavirus and West Nile virus [83,84], trematodes [85], and bacteria [86]. Recent evidence identified that wildlife reservoirs for zoonotic diseases increase their abundance in human-dominated ecosystems [87].

The introduction and spread of invasive alien species are recognized as leading causes of biodiversity loss [88,89]. Invasive species threaten biodiversity through different pathways including predation [90], competition [91], and disease transmission [92]. The use of ecosystems by humans is associated with local extinction of native species, the introduction of exotic species into new habitats, and a rise in the populations of species that are good reservoirs for zoonotic pathogens such as hantavirus reservoirs in the Americas [93].

### 4.5. Change in the Global Ocean

The global ocean covers 71% of the world’s surface, provides the majority of the oxygen we breathe, helps regulate climate, and moderates the risks of climate change. It provides food, employment, energy, health enhancement, and pharmaceutical and other products [94]. However, the capacity of the ocean to continue to provide these benefits is reducing, with carbon dioxide emissions increasing ocean acidification and with climate change increasing sea surface temperature and decreasing ocean oxygen levels, resulting in loss of habitat, changes to fish migration, decreased fish stocks, and changes to the nutritional value of some species [60,94]. Ninety percent of ocean fish stocks are either over or fully fished [95] and do not have the capacity to provide the recommended level of fish for healthy diets, increasing food insecurity and undernutrition [60].

Further, the ocean is being degraded, with up to 80% of marine pollution coming from the land [60]. For many low- and middle-income countries, this includes 90% of untreated wastewater and 70% of industrial waste [60]. These dispersal systems carry pathogens such as viruses, antibiotic microbial resistant bacteria, and a range of chemicals from industrial, agricultural, domestic, and urban sources (water run-off from cities, etc.) [96,97,98,99,100] and plastics have been found in all strata of the ocean [101].

These pollutants compound the negative impact of climate change and have direct health consequences, such as disease transmission and ingestion of toxic substances [102]. Future projections are mixed, with some suggesting seafood insecurity could increase, which could contribute to conflicts, especially in the Asia region [60,103]; however, Reference [104] suggests that improved global management of wild fisheries, along with global policy reform and technology improvements, could increase ocean-based yields by 36–74% by 2050.

### 4.6. Air Pollution

Air pollution–indoor and outdoor—is the world’s largest single environmental health risk, accounting for about 7 million deaths annually [29]; most of it stems from the energy produced by the combustion of fossil fuels. It was estimated that 85% of fine particulate air pollution is related to energy use [105]. At the same time, people in many low- and middle-income countries lack sufficient energy, with available energy sources such as biomass burning associated with many health effects [106]. About three billion people worldwide cook on open fires or use dirty and polluting fuels. Poverty and the use of solid fuels are inextricably linked with poor health, particularly for women and children.

The changing climate also affects the formation, dispersion, and transport of air pollutants. In general, higher temperature results in higher emission of gaseous pollutants and formation/photochemical reactions of secondary pollution [29]. For example, global ozone concentration increased in recent years, with concentrations expected to increase further with climate change. Moreover, changing weather patterns could affect pollutant levels; the consequences for acute and chronic health conditions require investigation. Another potential impact of climate change is an increase in biological aerosols; pollen and mold spores can cause allergic diseases such as asthma and allergic rhinitis [107].

### 4.7. Water and Sanitation Systems

By 2025, half of the world’s population will be living in water-stressed areas. It is expected that there will be at least 2 billion people utilizing a drinking water source that is contaminated with feces from improper sanitation. Contaminated drinking water is estimated to cause 502,000 diarrheal deaths per year [108]. Currently, 844 million people lack basic drinking water services [109]. Higher temperatures and more extreme, less predictable, weather conditions are anticipated to affect the availability and distribution of rainfall, snowmelt, river flows, and groundwater, and further, deteriorate water quality [110]. People in low-income communities, already the most vulnerable to any threats to water supply, are likely to be worst affected. Changes in water availability and quality, and consequently the use of wastewater, will impact health and food security [111,112] and refugee dynamics and political instability [110].

Chemical and microbial contaminants from wastewater during heavy rain events and floods can pollute water sources. In urban areas, untreated wastewater coming from industries and households may affect surface and groundwater quality [113]. Any decrease in water quality may further complicate the ability to adequately and effectively treat water sources [114,115]. In some rural areas, humans and agriculture share the same water sources, increasing the possibility of contamination. Heavy rain-related flooding and/or water overflow can increase contaminants coming from agriculture and soil run-off [116]. In China and many other regions, water scarcity can reduce agricultural productivity, leading to food insecurity and undernutrition [114,117].

Higher precipitation and flooding associated with climate variability and change can increase runoff, exacerbating the pollution of surface water and groundwater [118]. The negative impacts of warming temperatures on crop yields may increase chemical use in agricultural zones. The use of contaminated wastewater in agriculture presents a risk for farmers and consumer health [111,112,118].

SDG 6 calls for sanitation and hygiene for vulnerable populations, yet several low- and middle-income countries, particularly in Africa and Asia, lag in access to sanitation services [119]. Projected sanitation concerns with climate change include damage and loss of services from floods and reduced carrying capacity of waters receiving wastewater [120]. Key actions to reduce climate risks include integrating measures of climate resilience into water safety plans, improved accounting, and management of water resources [121]. Technologies for service delivery and changes in management models offer the potential to reduce risks, particularly in low-income settings.

## 5. Research Agenda to Protect and Promote Human in the Context of Global Environmental Changes

**Figure 3:**
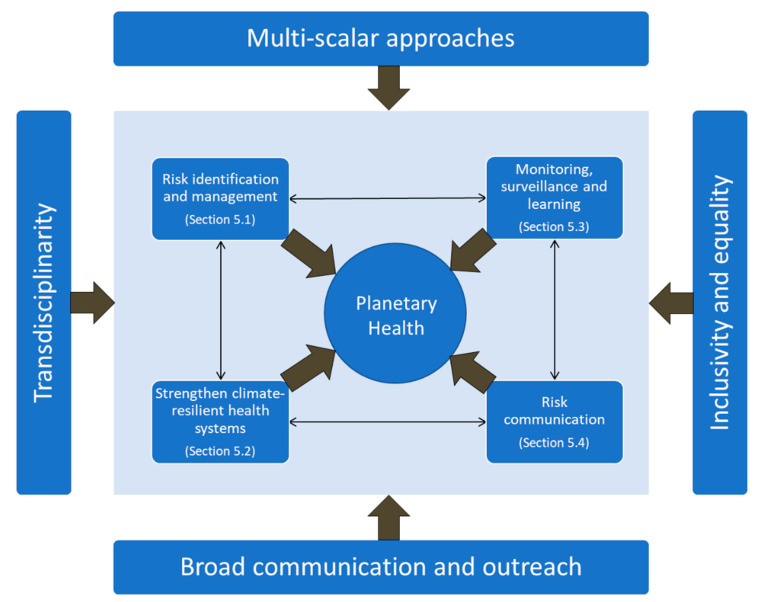
Conceptual framework for Planetary Health proposed for the application of the Health KAN research agenda.

A research agenda is elaborated in the following sections to address cross-cutting knowledge gaps to further understanding and management of the health risks of global environmental change according to the framework proposed in Figure 3. Inner boxes and their connectivity are discussed in Section 5.1, Section 5.2, Section 5.3, Section 5.4, and the outer boxes reflect approaches needed for the pathways toward sustainability as discussed in Section 6.

### 5.1. Risk Identification and Management

#### 5.1.1. Water, Hygiene, Sanitation, and Waste Management

Adapting water and sanitation systems to ongoing and projected socioeconomic and environmental changes is critical to better protect human health globally, with the aim of ensuring clean water, improved sanitation, and proper hygiene conditions over the coming decades. Research is needed to anticipate the likely impacts of warmer temperatures and changes in the hydrological cycle on drinking water quality and quantity [122,123].

There is a need for the re-design of sanitation infrastructure, adapted to water shortages and/or overflows. Safe toilets are important for human dignity, privacy, and physical security according to Resolution 70/169 [124]. The use of toilet facilities is indoctrinated into the social and cultural norms within societal structures; there is an urgent need for equitable access to toilet and sanitation facilities for all.

A paradigm shift is needed from waste being disposed of far away to resource recovery and reuse as a sustainable way of managing waste. Traditional waste and e-waste are key considerations for waste management that incorporate behavioral changes in consumption from individual to community levels.

Similar transitions are needed for sanitation, e.g., sanitation facilities, wastewater treatment, and fecal sludge management [125]. Projected sanitation concerns with climate change include damage and loss of services from floods and reduced carrying capacity of waters receiving wastewater [120]. Key actions to reduce climate risks include integrating measures of climate resilience into water safety plans, improved accounting, and management of water resources [121]. Technologies for service delivery and changes in management models offer the potential to reduce risks, particularly in low-income settings.

#### 5.1.2. Food Production and Consumption

Research is needed on the role of rising atmospheric concentrations of carbon dioxide, climate change, land-use change, and changing diets on the magnitude and pattern of food insecurity under a range of socioeconomic development pathways and assumptions of the extent and effectiveness of policies to reduce greenhouse gas emissions and to adapt to the consequences of a changing climate (including synergies and trade-offs). Research also is needed on solutions to address reductions in food quality from higher carbon dioxide concentrations, and food safety from the increasingly industrialized production practices.

Research is needed to understand the varied characteristics, drivers, and impacts of dietary preferences, changes, and transitions across geographies, cultures, and levels of economic development and urbanization. There is a need to project dietary changes under a range of possible futures, including the possible consequences for sustainability and planetary health. Policy options and practical solutions need to be developed and deployed to foster sustainable dietary transitions [63].

#### 5.1.3. Oceans

Given the continued dispersal of pollutants into the oceans, continued stress on wild fish stocks, and the lack of progress on SDG 14, it is urgent that social policy researchers investigate how our global governance can be improved [126]. Specifically, what is required to improve the monitoring and assessment of programs like The Global Programme of Action for the Protection of the Marine Environment from Land-based Activities and to identify and improve mechanisms such as targeted regulatory and institutional frameworks so as to improve access, markets, and supports for small-scale fishers. Finally, continued research is required to increase aquaculture and mariculture production in a way that is sustainable and socially inclusive.

#### 5.1.4. Extreme Weather Events and Climate Change

Transdisciplinary research is needed to further knowledge of extreme weather and climate events on health, including injuries and illnesses, infectious disease emergence and spread, food security, and mental health, and on healthcare institutions, including the costs of impacts. Research is needed to advance the practice of disaster risk management, preparedness, response, and communication, including through event forecasting and early warning systems. Research should evaluate the effectiveness of ecosystem and nature-based strategies, such as the protection of wetlands, coral reefs, and mangroves. Research also is needed to understand the effectiveness of social safety nets for reducing vulnerability to extreme events, and the effectiveness of adaptation strategies in reducing health risks, particularly in vulnerable communities and regions. Examples include spatial planning, such as zoning, that can limit an increase in risk by 25% to 45%; and flood-proofing measures at residential levels capable of reducing risk by 30% to 40%% [120]. Combining spatial zoning and floodproofing strategies could significantly reduce the overall increase in risk by 2030 by 40% to 60% [127]. Models are needed for future health risks and potential losses and damages to critical health infrastructure, including economic and societal costs of disaster preparedness and response under the Shared Socioeconomic Pathways (SSPs).

### 5.2. Strengthen Climate-Resilient Health Systems

Strengthening health systems to govern the risks of climate change, biodiversity loss, and land-use change would reduce present and future health burdens [128]. Generally, health systems prioritize the funding of curative, rather than preventive health. In many low- and middle-income countries, health infrastructure is already extended beyond its capacity to provide even fundamental health services security. Unless immediate and effective adaptation and mitigation solutions are implemented, climate change will continue to result in additional unmet demands for healthcare services and facilities and a growing disease burden [129].

The World Health Organization Operational Framework for building climate-resilient health systems outlines the ten components and associated processes for the health system to be prepared for the challenges and opportunities of a changing climate [130]. Core elements include developing a national health adaptation plan involving all sectors related to the social, economic, and political determinants of health; comprehensive and integrated health and environmental information systems to inform decisions, develop early warning systems, and provide policy recommendations; [131] and implementing, monitoring, and evaluating timely and effective interventions that address the needs of vulnerable and resource-poor groups [128,132,133]. Collaborations need to be established with national meteorological, hydrological, and environmental services to collect appropriate climate/weather/environmental data to facilitate managing and communicating climate-related health risks [134]. A global collaborative process should complement existing initiatives, such as the information being collected under the WHO/UNFCCC Health and Climate Change Country Profiles; the joint work program between WHO and the Convention on Biological Diversity (CBD); the Sustainable Healthy Urban Environments (SHUE) project; the Sendai Framework Monitor and Disaster Loss Database; and the Lancet Countdown on Health and Climate Change.

More than half of the climate footprint of health care comes from energy use, so mitigation and adaptation should include the use of technologies that reduce greenhouse gas emissions and environmental pollution and that increase facilities’ resilience to extreme weather events and other disasters [134]. Low-carbon healthcare includes considering building design and construction, investment in renewable energy and energy efficiency, sustainable healthcare waste management, transport and water consumption policies, and low-carbon procurement policies for pharmaceuticals, medical devices, other products, and supply chains.

Green infrastructure strategies, low emission public transport networks, and the promotion of active travel (walking and cycling) can improve health by preventing non-communicable diseases through reduced air pollution and reduced vehicular crashes. Green space also can increase physical activity, social and economic benefits, and improve cities’ resilience to extreme environmental events such as heatwaves (by mitigating the urban heat island effect) and extreme rainfall (by reducing surface run-off) [135].

Health care systems should adopt resilience strategies to strengthen their ability to cope with and recover from climate-related shocks and stresses and build the capacity of health professionals to prepare for and deal with the health impacts. This includes investments in building resilient health infrastructure and capacity development of human resources to use the climate information and applying it for improved health outcomes.

Research gaps related to resilience and climate smart health care include a better understanding of approaches to prevention, prediction, and preparedness; early warning systems; pathway analyses of climate emissions from health care; economic analysis of the costs and benefits of transitioning to climate-smart health care; and risk communication.

### 5.3. Monitoring, Surveillance, and Evaluation

Monitoring and evaluation of the flow of the resources, energy, and information are essential for adaptation. Simultaneously monitoring population and planetary health is critical for understanding the causal pathways between environmental parameters (including weather/climate, atmosphere, land use and crop yields, biodiversity) and the health and well-being of populations, taking into account the multiple drivers of adverse health outcomes. Monitoring can facilitate an action-oriented research plan to evaluate success.

Environmental data can come from field stations, satellites, surveys, etc. Other environmental and health sources include in situ sensors and smartphone-based geocoded personal data collection. Geographic information system tools can be useful for vulnerability mapping. A capacity-building component also may be needed, including professional development for staff in relevant disciplines, especially in low-income countries. Analysis of these data can lead to new insights about the environment–health relationships and to identify a range of possible interventions, including synergies and trade-offs with other programs.

Collective learning can be supported by creating an “Urban Brain” like that proposed in the Xiamen Call for Action [136]. Exploring appropriate ways of citizen participation in health monitoring may be valuable. Passive disease outbreak surveillance and reporting systems can be exploited and used with environmental and climate observations to assess and monitor disease outbreak patterns and trends globally.

### 5.4. Risk Communication

There is a need for effective and consistent strategies for communication and health promotion that increase the resilience of vulnerable communities and regions. A transdisciplinary approach brings together researchers, governments, NGOs, and businesses to work together to mitigate impacts and implement action plans. People need to know what health risks they could face due to global environmental changes and what actions they can take to protect their lives, livelihoods, and health, and how to contribute to the assessment and management of risk [137]. Tailored communication tools are needed to meet the specific needs of different disadvantaged groups, such as migrant communities. Risk communication efforts during short-term extreme weather events appear to be more effective in motivating adaptive changes than communication about health risks or climate change. Effective communications require close collaboration between public health professionals, scientists, and communicators, particularly around what the future could bring with respect to, for example, pathogen emergence and spread.

There is a research-practice gap with respect to institutional change in the healthcare sector, including how to modify the patterns of knowledge, practice, and values that make up comprehensive health care [138]. Health professionals are ideal advocates for group action on adaptation and mitigation policies and can play a vital role in public education and awareness through the timely exchange of information, advice, and opinions among experts, community leaders, or officials and the people most at risk [139]. More innovative, interdisciplinary, people-centered, participatory research can foster crucial trust and transform people’s perception of risk and their risk-reduction behaviors and communicate prevention [137].

## 6. Discussion: Pathways toward Sustainability

This review details a comprehensive research agenda to inform, develop, implement, and evaluate interventions to improve sustainable development for planetary health and human well-being. Scientific investigation should go beyond impact assessments. Adaptation strategies and transformation pathways need to be identified, implemented, and monitored. Innovative energy strategies and technologies offer promise for health, equity, and sustainable development [140,141]; well-crafted policies can reduce greenhouse gas and short-lived climate pollutant emissions whilst yielding co-benefits across multiple domains [142,143].

Evaluations are needed of the effectiveness and benefit–cost ratio of mitigation and adaptation on reducing greenhouse gas emissions, air pollution, and health benefits [144]. The positive and negative health and wellbeing effects need to be evaluated of many adaptation measures such as active transportation, greening, particularly in urban settings, smart buildings, personal healthcare with information communication technology (ICT) transition, and other innovations.

Preventing, preparing for, and responding to these risks require multisectoral and multidisciplinary approaches that engage different sectors and actors such as governments, business, and civil society along with researchers and scientific bodies to work together on a common agenda of prevention, adaptation, and mitigation of risks and impact [145]. This necessitates that climate and disaster risk reduction must be an integral part of national health policies and interventions to ensure the resilience of the health sector.

Transdisciplinary, solutions-oriented innovative research across spatial and temporal scales is needed to identify health-promoting, cost-effective patterns of development to realize co-benefits for health, environment, and economy, and to identify effective policy and governance strategies to shape healthy and sustainable settings that manage short- and long-term risks [146,147]. To promote sustainable transitions, this research should be committed to inclusivity, equity, co-production, and scientific rigor. We recommend the research priorities be pursued through approaches that are (as indicated in Figure 3):

- **Transdisciplinary**, inviting stakeholders to co-design research and implement interventions [148,149]. Transdisciplinary research into reducing vulnerability and enhancing resilience should cut across several domains, including (but not limited to) the built environment, natural ecosystems and biodiversity, social and cultural contexts, economics, and governance. Such research could inform policymaking for all relevant sectors and actors including governments, businesses, and civil society organizations. This approach could also inform mainstreaming health into national strategies and action plans to implement relevant multi-hazard international frameworks (e.g., the New Urban Agenda, the Sendai Framework for Disaster Risk Reduction and the Paris Agreement for Climate, and the Biodiversity Framework), and promoting international collaborations, information sharing, and capacity building. Moreover, the SDGs provide a comprehensive framework for a more integrated and preventive risk reduction approach in health.
- **Multi-scalar**, understanding historical patterns of planetary health impacts to establish causal relationships from the local to the global and projecting the possible magnitude and pattern of future risks under different development pathways. This knowledge is necessary for designing, implementing, and monitoring solutions to improve population health in the short-, mid-, and long-term.
- **Inclusive and equitable** by being responsive to intersectional aspects such as gender, age, social status, and ethnicity, in how research is conducted and in how solutions are developed. The intergenerational justice impacts of global environmental health research are considered. Equitable research includes searching for funding structures that enable South-South and North-South cooperation and that aim for diverse representation in research projects.
- **Communicated broadly**, sharing newly generated knowledge with different audiences. This includes dissemination beyond academia and experimenting with creative communication approaches to inform policy development in adaptation and mitigation.

In a post-pandemic world, a critical research need is to re-visit the SDG targets and indicators to determine if revisions/re-design would strengthen linkages with risk drivers and would facilitate urgent action to combat climate change and its impacts. Partnerships between the research community and businesses, and state and non-state actors, are needed to explore the contribution of non-marketed ecosystem services (e.g., pollination, microclimatic stabilization, water purification) to planetary health. Another critical research need is to explore how the international community can support commitments toward the health-related targets through investment choices and resource flows across borders. Country-specific research needs include those that can contribute to mainstreaming the costs and benefits of the economic and social value of good health and well-being into decision-making as economies chart their recovery paths. The research agenda for human and planetary health is broad and will require integrated systems-based thinking to achieve a new vision of sustainability: from the perspective of health, sustainability becomes a more integrated, holistic concept and the separate SDG boxes (many of which we refer to in the agenda) become more interconnected. Bringing human and planetary health together makes clear that ‘health’ is a shared systemic concept for humans and any other living systems.

The Future Earth Health KAN aims to generate the knowledge needed to take robust policy decisions and to build, strengthen, and connect national, regional, and global sustainability research communities and reinforce links with other stakeholders that have an interest in and influence on pathways to the SDGs [150].

## 7. Conclusions

Human and planetary health are intimately interconnected with socioeconomic transitions and environmental change. The comprehensive research agenda presented here, and the Future Earth Health Knowledge Action Network meeting where it was initiated, was developed to influence research efforts worldwide by identifying critical knowledge gaps and needs for transdisciplinary efforts to generate the understanding needed to underpin robust policy decisions for human and planetary health. The research agenda identifies four priority research themes to sustainably protect and promote planetary health and human well-being: (a) risk identification and management (including related to water, hygiene, sanitation, and waste management; food production and consumption; oceans; and extreme weather events and climate change); (b) strengthening climate-resilient health systems; (c) monitoring, surveillance, and evaluation; and (d) risk communication. These should be tackled with research approaches that are transdisciplinary, multi-scalar, inclusive, equitable, and broadly communicated. Knowledge generated from the outlined research can inform policies to build, strengthen, and connect national, regional, and global sustainability research communities. It also can promote collaborations with other stakeholders with an interest in and ability to influence resilient and sustainable development pathways to achieve human and planetary health.

## Appendix A

The Future Earth Health Knowledge Action Network (Health KAN) was established to support and enable solution-driven transdisciplinary research and action to improve understanding of how global and regional environmental changes can affect population health and health systems, and of effective and efficient solutions to maintain and improve planetary health and human well-being. The Health KAN provides a forum for inter- and transdisciplinary, multi-country, large-scale research and action across temporal and spatial scales that will contribute scientific knowledge to inform just and equitable societal transitions to a more sustainable future. The Health KAN presents a unique opportunity to bring together experts from across the globe from all relevant disciplines, NGOs, and stakeholder organizations, to collectively broaden and strengthen the impact of individual efforts that can achieve much-needed progress to promote and protect population health in the Anthropocene and contribute to the Future Earth Global Strategy to facilitate research and innovation, build and mobilize networks, and shape the global narrative. Future Earth was officially announced in 2012 at the UN Conference on Sustainable Development, with the goal of *accelerating transformations to global sustainability through research and innovation*, and became fully operational at the end of 2015 [9]. Key entities of Future Earth structure are Knowledge Action Networks (KAN). Following three years of preparations and development, the Health KAN was officially launched in May 2019 at an event that included experts in planetary health, funders, representatives of a range of international organizations, and policymakers. The mission of the Health KAN is to *support and enable solution-driven transdisciplinary health research, and to improve the understanding of linkages between health and the environment*. This global, bottom-up initiative aims to convene diverse groups of stakeholders to inform policy dialogue and help develop a shared vision among scientists and stakeholders regarding the characteristics of pathways that will lead to sustainability and to the achievement of the related SDGs. The Health KAN focuses on promoting holistic solutions for planetary health and human well-being with all stakeholders.

## Appendix B

**Table A1:** Participants of the Health KAN launch event.

No.	Family Name	Name (s)	Affiliation
1	Anyamba	Assaf	USRA and NASA Goddard Space Flight Center
2	Bi	Peng	University of Adelaide, Australia
3	Boeckmann	Melanie	Bielefeld University, Research Group Environment and Health, Germany
4	Bowen	Kathryn	Australian National University/University of Melbourne
5	Capon	Anthony	University of Sydney (Future Earth Health KAN Co-chair)
6	Charles-Ayinde	Makyba K S	AAAS, Future Earth, National Science Foundation
7	Cheong	Hae-Kwan	Sungkyunkwan University School of Medicine
8	Cissé	Guéladio	Swiss Tropical and Public Health Institute
9	Dasgupta	Purnamita	Institute of Economic Growth, Delhi, India
10	Daszak	Peter	EcoHealth Alliance (Future Earth GRP oneHEALTH)
11	Ebi	Kristie L.	University of Washington (Future Earth Health KAN Co-chair)
12	Gasparatos	Alexandros	The University of Tokyo
13	Hashizume	Masahiro	Nagasaki University
14	Honda	Yasushi	University of Tsukuba
15	Javadi	Firouzeh	Institute of Decision Science for a Sustainable Society, Kyushu University
16	Kanbara	Sakiko	University of Kochi
17	Kasuga	Fumiko	Future Earth
18	Kim	Ho	Seoul National University
19	Koné	Brama	Centre Suisse de Recherches Scientifiques en Côte d’Ivoire & Université péléforo Gon Coulibaly of Korhogo
20	Kovats	Sari	London School of Hygiene and Tropical Medicine
21	Kusuma	Parama Tirta Wulandari Wening	Indonesian Institute of Sciences, Bureau for Financial Planning
22	Lee	Shihyu	Research Center for Environmental Changes
23	Lee	Chia-Hsing Jeffery	Center for Sustainability Science, Academia Sinica, and Future Earth-Taipei
24	Lung	Shih-Chun Candice	Research Center for Environmental Changes, Academia Sinica
25	Mallee	Hein	Regional Centre for Future Earth in Asia, Research Institute for Humanity and Nature
26	Maycock	Bruce	Curtin University
27	Morse	Andy	University of Liverpool
28	Murakami	Takahiro	Institution of Decision Science for a Sustainable Society, Kyushu University
29	Mustapha	Adetoun	Nigerian Institute for Medical Research; Imperial College London and International Society for Environmental Epidemiology, Africa Chapter
30	Nagashima	Miki	Malaria No More Japan
31	Onofre	Edwin A. Merilles Jr.	The Pacific Community (SPC)
32	Pongsiri	Montira	Oxford University
33	Raivio	Kari Olavi	University of Helsinki
34	Sioen	Giles B.	Future Earth, Institute for Future Initiatives, The University of Tokyo, The United Nations University Institute for the Advanced Study of Sustainability
35	Sobral	Bruno Walther Santos	One Health Institute, Colorado State University
36	Suzán	Gerardo	National Autonomous University of Mexico UNAM
37	Tanimoto	Hiroshi	National Institute for Environmental Studies
38	Wannous	Chadia	TASW Network, Health KAN Development Team
39	Watanabe	Chiho	National Institute for Environmental Studies
40	Wegerdt	Johannah	Green Climate Fund
41	Wu	Pei-Chih, Peggy	Chang Jung Christian University
42	Yamauchi	Taro	Hokkaido University/Research Institute for Humanity and Nature
